# Infection prevention in catheter-interventional treatment of children and adults

**DOI:** 10.3205/dgkh000385

**Published:** 2021-03-24

**Authors:** Heike Schneider, Thomas Paul

**Affiliations:** 1 Department of Pediatric Cardiology, Intensive Care Medicine and Neonatology, University Medical Center Göttingen, Göttingen, Germany

**Keywords:** interventional pediatric cardiology, cardiology, infection prevention

## Abstract

Catheter-interventional treatment is a growing field in pediatric cardiology and cardiology, replacing an increasing number of operations. This article provides an overview of the general practice of hygienic measures and antimicrobial prophylaxis in the cardiac catheterization laboratory to prevent post-procedural infection, particularly if foreign material is utilized.

## Zusammenfassung

Die katheterinterventionelle Behandlung ist ein stetig wachsendes Feld der Kinderkardiologie und Kardiologie, das zunehmend Operationen ersetzt. Dieser Artikel gibt einen Überblick über die allgemeine Handhabung von hygienischen Maßnahmen und antimikrobieller Prophylaxe im Herzkatheterlabor, um postprozedurale Infektionen zu verhindern, insbesondere wenn Fremdmaterial eingesetzt wird.

## Introduction

Within the past 20 years, significant progress has been achieved in catheter-interventional treatment of children and adults with congenital cardiac heart disease as well as in patients with acquired cardiac lesions.

These achievements were made possible by the development of special cardiac implants or devices primarily designed for treatment of congenital heart defects and valve lesions, which have subsequently been introduced into clinical practice for patients of all ages. 

Nowadays, modifications of these devices are used in adults to close persistent foramen ovale to prevent embolic stroke.

In contrast, vascular stents were primarily developed to treat peripheral vascular stenosis and stenotic coronary arteries in the adult population, but have also been used in children with vessel stenosis, such as coarctation of the aorta or peripheral pulmonary artery stenosis, and even to keep a ductus arteriosus patent in infants with either pulmonary or systemic duct-dependent circulations.

Today, these developments allow minimally invasive treatment of a large number of congenital and acquired heart defects by catheter interventions. A significant proportion of cardiac surgery is already being replaced by (and in the future will even more commonly replace) catheter interventions; this enables patients to be mobilized earlier and start rehabilitation sooner, with less surgical trauma and pain. Limitations, such as the small size of children, and associated problems; such as vascular access, will be overcome via combined surgical and interventional hybrid procedures.

## Examples of catheter-interventional treatment options for congenital and acquired heart defects

### Balloon valvuloplasty of stenotic semilunar valves

Balloon dilation is the main treatment option for valvar pulmonary stenosis, with good long-term outcome for patients of all age groups. The rate of re-stenosis is low. Complications are rare and are usually limited to vascular access sites [1]. Aortic valve stenosis in children can also be treated well by valvuloplasty and a significant reduction of the transvalvular gradient can be achieved [2]. These procedures can be performed in the cardiac catheterization laboratory without additional measures regarding hygiene standards. Pre-procedural antibiotic prophylaxis is not required [3].

### Electrophysiological studies and catheter ablation

Nowadays a large number of cases of supraventricular and ventricular tachycardia can be effectively treated and cured in adults as well as in children by means of radiofrequency or cryothermal catheter ablation [4]. These procedures can be performed in the cardiac catheter or electrophysiological laboratory under aseptic conditions and without additional hygiene measures. Pre-procedural antibiotic prophylaxis is not required [3].

### Catheter-interventional procedures with implantation of non-biological foreign material and implantation of cardiac pacemakers and internal cardioverter/defibrillators (ICD)

Higher requirements for hygienic conditions exist for these procedures to prevent peri-interventional infection or endocarditis. Ideally, these interventions should be performed in specially designed hybrid suites or in an operating room, where surgical procedures can be performed under aseptic conditions with the option of utilizing high-quality fluoroscopy and angiography.

In the conventional cardiac catheterization laboratory, a mobile laminar airflow system positioned at the aseptic catheterization table or operation field (2020 communication with A Kramer; unreferenced, see “Notes”) can be employed as an alternative to prevent contamination of the device by airborne microorganisms.

During implantation, peri-interventional prophylactic antibiotic prophylaxis with a single dose of cefazolin or cefuroxime is recommended; data concering infection-prevention effectiveness, however, are currently unavailable [3], [5], [6]. After the complete closure of a heart defect, endocarditis prophylaxis (as secondary prophylaxis, e.g., before a dental procedure) is recommended for 6 months, until complete endothelialization of the device is achieved [3], [7]. Further recommendations are given in the following according to the particular cardiac defect.

### Patent ductus arteriosus

If the ductus arteriosus remains patent, a left-to-right shunt exists that can result in left ventricular volume overload, depending on diameter and length of the ductus, with the risk of developing pulmonary arterial hypertension. Today, the method of choice after the neonatal period is to close the ductus by catheter intervention. Several different occluder systems are available for closing the ductus, such as occluding coils and specially-designed double-disc occluder systems made of nitinol. Almost any ductus independent of size and length can be treated in the catheterization laboratory with these occluders [8]. 

Access to the ductus can be gained either in an antegrade fashion via the femoral vein through the right atrium, right ventricle and the pulmonary trunk, or in a retrograde approach via the aorta. Size of the child and morphology of the ductus are important parameters to consider when selecting the ideal device. A residual shunt after device occlusion is infrequent, and a secondary intervention is rarely necessary. Peri-interventional antibiotic prophylaxis with cefuroxime is recommended [3]. Potential complications include embolization of the device, obstruction of the aorta or the left pulmonary artery, vascular injury, and hemolysis, which is usually transient.

Interventional duct occlusion in preterm infants and neonates has been decribed. In this age group, however, the procedure is associated with a significantly higher rate of complications. After complete closure of the ductus without residual shunting, endocarditis prophylaxis is recommended for 6 months [7], [8].

### Atrial septal defects

A tissue defect within the atrial septum is present. The atrial septal defect of the secundum type is located centrally within the oval fossa. A left-to-right shunt exists between the atria, resulting in volume overload of the right atrium, right ventricle, and the pulmonary circulation. As typical sequelae, pulmonary arterial hypertension can develop in early adulthood with secondary decrease of right ventricular function. In addition, types of supraventricular tachycardia, such as atrial flutter or atrial fi-brillation, may occur.

**Figure 1:**
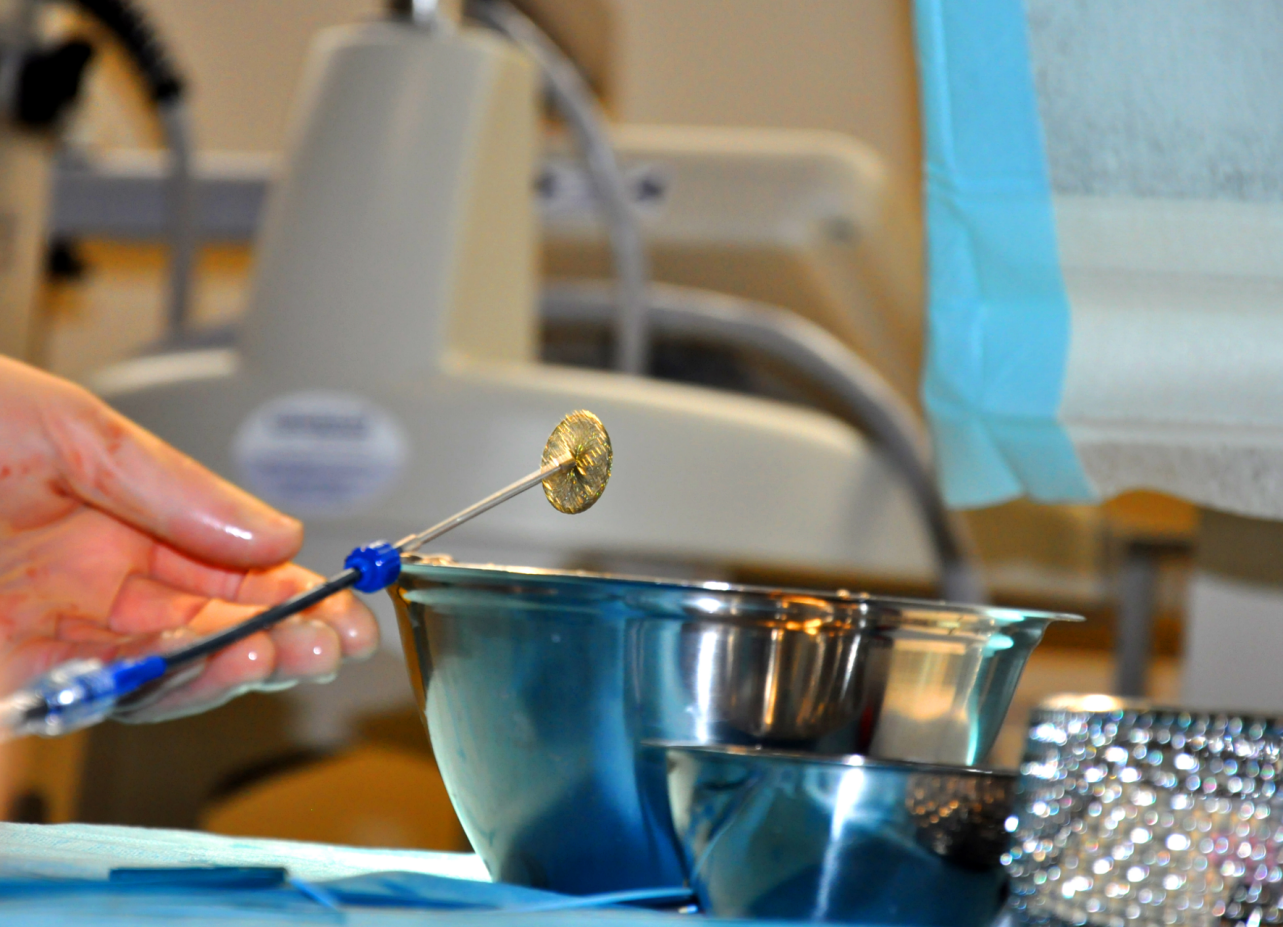
A double-disc device (Occlutech ASD Occluder, Occlutech, Jena, Germany) for closure of an atrial septal defect is already screwed onto the delivery system.

The method of choice today to close an atrial septal defect of the secundum type is interventional closure with a double-disc device (Figure 1 (Fig. 1)). There is a wide array of occluders on the market with a diversity of designs and different sizes from several manufacturers [9]. 

**Figure 2:**
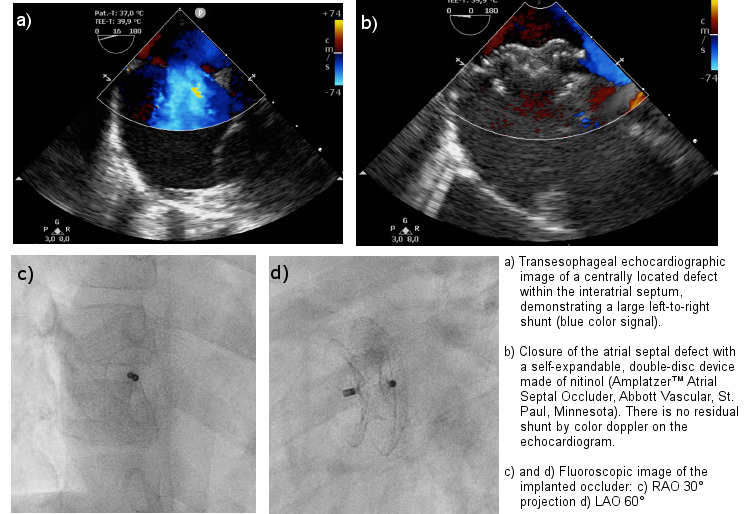
Catheter-interventional closure of an atrial septal defect of the secundum type in a 6-year-old boy

In children, the procedure is performed often under general anesthesia, as continuous monitoring of the pro-ce-dure by transesophageal echocardiography is used (Figure 2 (Fig. 2)). Monitoring by transthoracic echocardiographic guidance under sedation is the alternative. Complications are rare and include device embolization, cardiac perforation, thromboembolic events, air embolization as well as supraventricular tachyarrhythmias. Peri-interventional antibiotic prophylaxis with cefuroxime is recommended [3]. 

After the procedure. endocarditis prophylaxis is recommended for 6 months, until complete endothelialisation of the device is achieved. In addition, anti-platelet therapy with aspirin at a low dose of 2–5 mg/kg is advised to prevent thrombus formation on the occluder. For adolescents and adults, dual antiplatelet therapy with aspirin 100 mg and clopidogrel 75 mg is administered for 6 to 12 months [9]. 

### Ventricular septal defects

The ventricular septal defect is the most common congenital heart defect. Ventricular septal defetcs can occur alone or in combination with other congenital heart defects. A defect in the perimembranous region of the interventricular septum is the most prevalent manifestation. A left-to-right shunt exists at the ventricular level; its quantity depends on the size of the defect and the level of the pulmonary vascular resistance. In general, increased pulmonary blood flow with volume overload of the left atrium and ventricle is present. If left untreated, large defects can lead to pulmonary arterial hypertension within the first year of life. Irreversible remodeling of the pulmonary arteries with a progressive increase in vascular resistance can result in reversal shunting via the ventricular defect (Eisenmenger reaction) [10]. In general, ventricular septal defects can be treated by catheter intervention. Limitations mainly include the size of the child and the risk of AV block in perimembranous ventricular septal defects in children <20 kg. As in atrial septal defects, peri-interventional antibiotic prophylaxis with cefuroxime is administered, followed by 6 months of endocardi-tis prophylaxis as well as antiplatelet therapy with aspirin at a dose between 2 to 5 mg/kg to prevent apposition of thrombi on the occluder. For adolescents and adults, a dual anitplatelet regimen consisting of aspirin 100 mg and clopidogrel 75 mg for 6–12 months is recommended [10]. 

### Percutaneous pulmonary valve implantation

In patients with complex congenital heart defects who underwent surgery including a conduit between the right ventricle and the pulmonary artery trunk, degenerative processes often occur that lead to significant pulmonary regurgitation and/or obstruction of the right ventricular outflow tract. 

**Figure 3:**
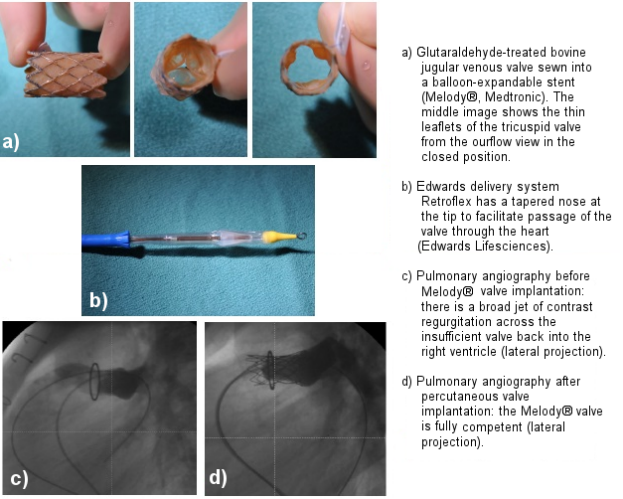
Percutaneous pulmonary valve implantation in a 17-year-old adolescent after surgical implantation of a 22-mm bioprosthetic valved Hancock® conduit (Medtronic) in a patient previously operated on for tetralogy of Fallot and pulmonary atresia: degenerative changes of the conduit ten years after his last open-heart surgery led to significant pulmonary regurgitation and stenosis, resulting in the indication for pulmonary valve implantation.

As an alternative to a surgical conduit replacement, a catheter-interventional procedure has been developed. A bovine jugular venous valve, treated with glutaraldehyde, is sewn into a balloon-expandable valve (Melody^®^, Medtronic, Minneapolis, Minnesota) (Figure 3 (Fig. 3)). The valved stent can be implanted into the failing conduit with a 22 F delivery system. The Melody^®^ valve has already been implanted successfully in >12,000 patients worldwide. Initial limitations included the size of the conduit ≤22 mm and a body weight >30 kg. Today, implantation in smaller children is technically feasable with application of a combined catheter-interventional and hybrid pediatric-cardiac-surgical procedure, even in children with a body weight of 10 kg [11]. For larger conduits, native or patched right ventricular outflow tracts, Edwards Sapien valves (Edwards, Lifescience, Irvine, California), originally developed for the calcified aortic valve of the elderly, are increasingly used in the pulmonary position in patients with operated congenital heart disease requiring a smaller delivery system (Figure 3 (Fig. 3)). Large self-expanding valves are nearing clinical approval. The implantation is performed under strict aseptic conditions. For implantation, peri-interventional antibiotic prophylaxis with cefazolin or cefuroxime is recommended; at least 3 doses are typically administered. The incidence of infection or endocardits after percutaneous pulmonary valve implantation has been reported to be approximately 3% per patient-year [12]. Uniform protocols, however, are lacking [3], [5], [6]. After valve implantation, life-long endocarditis prophylaxis is recommended [7], and in general antiplatelet therapy with aspirin 2–5 mg/kg (maximum 100 mg) for at least one year.

### Transcatheter aortic valve implantation (TAVI)

The most common cardiac lesion in adults is degenerative aortic valve stenosis, a disease of the elderly population. To minimize the significant risk of open-heart surgery for aortic valve replacement, due to the patients’ advanced age and often associated multimorbidity, transcatheter aortic valve implantation has been developed. Similar to the percutaneous pulmomary valve implantation, a balloon- expandable or a self-expanding valve mounted on a stent can be implanted either transfemorally or transapically (in the hybrid suite). This procedure has been shown to be superior to pharmaceutical therapy in high-risk patients. Recently, in patients with an intermediate operative risk, transfemoral aortic valve implantation has not been found to be inferior to surgery [13]. Peri-interventional antibiotic prophylaxis is also performed and supported by the ESC, with cephalosporin monotherapy being used most frequently [14], [15].

### Edge-to-Edge-Repair of AV valves

Severe regurgitation of the atrioventricular valves in el-derly patients is associated with a poor prognosis. Therefore, minimally invasive procedures to reconstruct the mitral valve have been developed. Using the Mitra-Clip^®^ system (Abbott Vascular, Abbott Park, Illinois), catheter interventional ‘edge-to-edge- repair’ can be performed for severe mitral regurgitation. During the procedure, dehiscent valve leaflets are clippped together. Again, peri-operative antibiotic prophylaxis is recommended for this procedure [15]. Recently, several other system, e.g., the Cardioband™ Mitral System and Cardioband™ Tricuspid Reconstruction System or the Pascal™ system (Edwards Lifesciences, Irvine California), have been developed for treatment of atrioventricular valve regurgitation. In the near future, transcatheter valves are expected to enter the market, targeting atrio-ventricular valve implantation.

### Hybrid procedures

**Figure 4:**
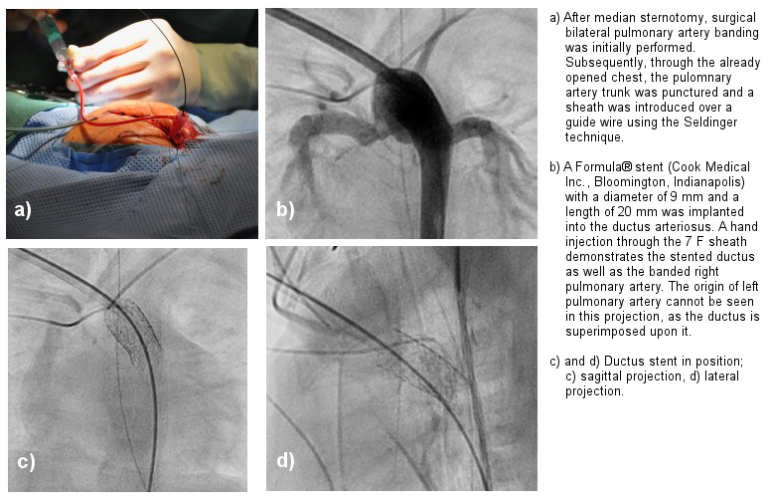
Combined pediatric-cardiac-surgical and catheter-interventional procedure in a hypotrophic newborn baby (body weight 2 kg) with hypopolastic left heart syndrome

Hybrid procedures are performed in close collaboration between cardiologists and cardiac surgeons, to overcome limitations of vascular access to the heart, to expands the potential of interventional therapy, and reduce the risk of the procedure (Figure 4 (Fig. 4)). These procedures should be performed in a specially designed hybrid suite to meet the hygienic standards of the operating room. For these procedures, peri-procedural antibiotic prophylaxis is also recommended [5], [6]. 

## Notes

### Competing interests

The authors declare that they have no competing interests.

### Acknowledgement

Alternatives were discussted verbally in the chapter “cardioverter/defibrillators (ICD)”:

Heike Schneider, Thomas Paul (Department of Pediatric Cardiology, Intensive Care Medicine and Neonatology, University Medical Center Göttingen, Germany). Conversation with: Prof. Dr. med. Axel Kramer (Institute of Hygiene and Environmental Medicine, University Medicine Greifswald, Germany). 2020 Dec 09.
